# Fast Human Detection for Intelligent Monitoring Using Surveillance Visible Sensors

**DOI:** 10.3390/s141121247

**Published:** 2014-11-11

**Authors:** Byoung Chul Ko, Mira Jeong, JaeYeal Nam

**Affiliations:** Department of Computer Engineering, Keimyung University, Sindang-dong, Dalseo-gu, Daegu 704-701, Korea; E-Mails: jeongmr@kmu.ac.kr (M.J.); jynam@kmu.ac.kr (J.N.)

**Keywords:** human detection, Hough windows map, adaptive ROI, divide-and-conquer, CaRF

## Abstract

Human detection using visible surveillance sensors is an important and challenging work for intruder detection and safety management. The biggest barrier of real-time human detection is the computational time required for dense image scaling and scanning windows extracted from an entire image. This paper proposes fast human detection by selecting optimal levels of image scale using each level's adaptive region-of-interest (ROI). To estimate the image-scaling level, we generate a Hough windows map (HWM) and select a few optimal image scales based on the strength of the HWM and the divide-and-conquer algorithm. Furthermore, adaptive ROIs are arranged per image scale to provide a different search area. We employ a cascade random forests classifier to separate candidate windows into human and nonhuman classes. The proposed algorithm has been successfully applied to real-world surveillance video sequences, and its detection accuracy and computational speed show a better performance than those of other related methods.

## 1. Introduction

Although human detection is an essential work for several computer vision applications such as human tracking, gesture recognition, action recognition, and video surveillance, the computational time required for human detection has been a significant burden for real-time processing. The improvement in speed of human detection has been studied in the following three ways:

- Reducing the overall number of feature computations [1,2].
- Reducing the time required to create the data structure for a block.
- Reducing the amount of image scaling and the number of search regions [3–5,6].

A popular human detection method is making a global human model using the histogram of oriented gradient (HOG) features with a sliding window and a finely multi-scale image pyramid [7]. However, a multi-scale image pyramid requires frequent image scaling, and the sliding windows should be applied at each scale for human detection.

To reduce the computational cost on the scaling of an image, Benenson *et al.* [3] presented a fast pedestrian detector running at over 100 fps on a single CPU + GPU enabled desktop computer. The core novelties of this approach are reverting the human detector of Dollár *et al.* [4] to avoid resizing the input image at multiple scales, and using a recent method to quickly access the geometric information from stereo images. Although this method shows a higher computational speed, it has the following limitations: (1) the detection performance was not significantly improved compared to conventional methods; (2) the CPU with should be used along with a GPU; and (3) the computational speed for a monocular image is about 85 fps slower than for stereo images. Dollár *et al.* [4] proposed a hybrid approach that uses a sparsely sampled image pyramid to approximate features at intermediate scales. The key insight of this method is that the feature responses computed at a single scale can be used to approximate the feature responses at similar scales. However, Dollár *et al.* did not describe in detail how to restrict the range of the image scale. Liang *et al.* [5] proposed a pedestrian detection method based on multi-scale scanning by exploiting the size information of the current region to avoid useless scales. However, because it uses the background subtraction model to reduce the range of the candidate regions, it is not applied to image sequences captured from a moving camera. Tang *et al.* [2] proposed a pedestrian detection method combining random forest and dominant orientation templates to improve the run-time speed. However, this method uses only features other than the image-scaling level for speed optimization, and therefore requires a reduction of the image-scaling level to obtain an additional speed-up.

Bae *et al.* [6] proposed using not only the image-scaling level by estimating the perspective of the image, but also the region-of-interest (ROI) for searching the area of a scaled image. However, this method does not include a way to determine the overlapping scale factors for the ROI according to the level of image scaling.

In addition, many classification methods have been proposed to reduce the computational time for human detection. Cascade AdaBoost [8] is a representative cascade strategy for human detection, and can reject most negative sliding windows during the early stages of the cascade steps. Cascade of random forests (CaRF) [9] is a three-level cascade of random forests that combines a series of random forest classifiers into a filter chain.

To reduce the computational complexity with high detection accuracy, we propose a Hough windows map (HWM) for determining the levels of image scaling, and an adaptive ROI algorithm for providing a different search area for each image scale. Moreover, we use CaRF with low-dimensional Haar-like features and oriented center symmetric-local binary patterns (OCS-LBP) [10] to verify a human region, instead of a conventional support vector machine [5,6] or cascade AdaBoost [8].

## 2. Estimation of Image-Scaling Level and Adaptive ROI

**Figure 1.**
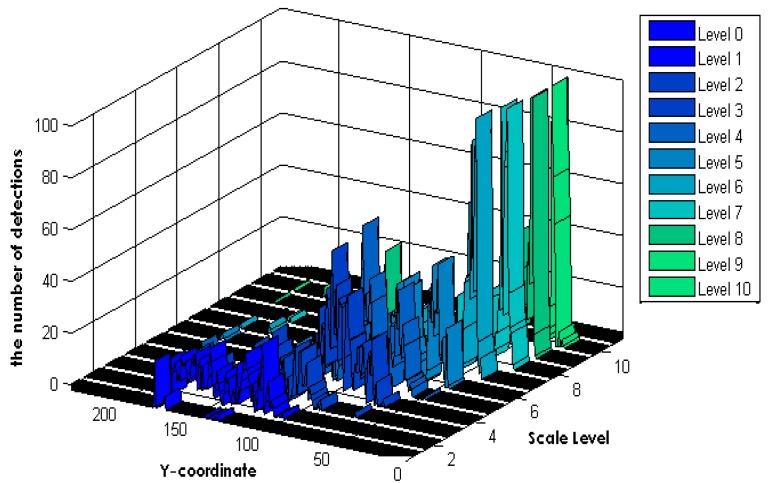
Accumulated Hough windows map information based on voting detection location. Detection locations are detected by applying naïve human detection [7] to eleven densely sampled levels of scale.

The proposed method assumes that the surveillance camera has a perspective view because most surveillance cameras are installed at a high location. To generate an HWM, we applied a naïve human detection algorithm [7] to a densely sampled image pyramid, and voting frequency of the HWM according to the Y-coordinate and scale level of the detection, as shown in Figure 1.

Because the main purpose of our algorithm is human detection in a perspective image captured from a surveillance camera, we assume that the smallest sized human should be detectable when the image is up-sampled to double its original size, and the largest sized human should be detectable when an image is down-sampled to half its original size. Therefore, we conduct image sampling to a test image at a ratio of 1:0.5 to 1:1.5 by densely increasing the scaling ratio (0.1) to determine the scaling level. In general, a template-matching algorithm is capable of comparing the similarities among different object sizes, even when there is little difference in size between the object model and the candidate object region [11].

The methods used for selecting the levels of image scaling and the adaptive ROI are summarized in Algorithm 1.



**Algorithm 1** Image scaling and adaptive ROI
R: a set of vectors [scaling level, ROI size]1. Apply N scaling levels to the test image with ratios of 1:0.5 to 1:1.5 by densely increasing the scaling ratio (0.1).2. Conduct dense human detection for N scaling images.3. Apply detection frequency voting of scaling level *i* for the HWM.4. HWM is divided into HWM_Left_ and HWM_Right_ equally using a divide-and-conquer algorithm. //call recursive function5. *Divide_Conquer_function*(R, HWM_Left_). *Divide_Conquer_function*(R, HWM_Right_).***Divide_Conquer_function*** (R, HWMs){ Accumulate all voting values of the HWM_*i*_ line by line; Find the maximum voting value among the accumulated HWM*_i_*; Choose one HWM having a maximum voting value with its Y- coordinate (*Max_y_*); Estimate a threshold T: 0.2 × (sum of voting value of HWM); If(maximum voting value of level *i* < T) //stop condition {  HWM_ROI_ = Adaptive_ROI(HWM, *Max_y_*, T);  Assign a vector [scaling level of HWM, HWM_ROI_] to R;  Return; //stop dividing } Else{  Divide HWM into HWM_Left_ and HWM_Right_;  *Divide_Conquer_function* (R, HWM_Left_); // call recursive function  *Divide_Conquer_function* (R, HWM _Right_); // call recursive function  }}***Adaptive_ROI*** (HWM, *Max_y_*, T){ Initial size of W: 0.3 × height of the scaled image Establish the initial ROI region centered at *Max_y_* with the initial size of W
(1)${\text{HWM}}_{\text{ROI}}=\{\left(x,y\right)|{\text{Max}}_{y}-\frac{W}{2}<\text{ROI}<{\text{Max}}_{y}+\frac{W}{2}\}$ Repeat  Sum (SUM) the voting value of HWM_ROI_;  If (SUM < T) expanding HWM_ROI_ by increasing W = W + 1;  Else break; Return the final region of HWM_ROI_ estimated by *Max_y_* and increased W;}


here, T (T > 0) and W (W > 0) are the control parameters, large values of which create even smaller scaling levels and a larger ROI size, whereas small values generate fine scaling levels and a smaller ROI size. In this paper, we set the initial values of T and W based on several experiments.

## 3. Cascade Random Forest for Human Classification

### 3.1. Feature Extraction

**Figure 2.**
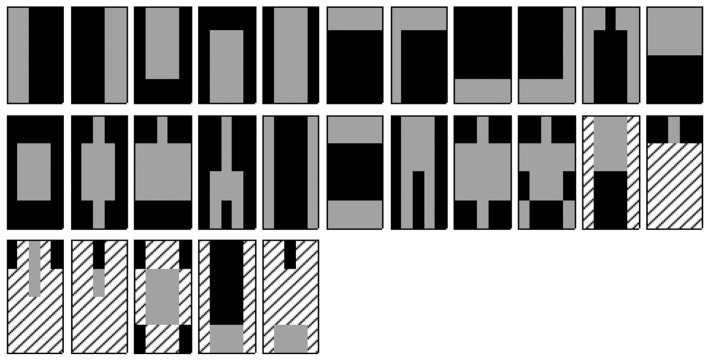
Twenty-seven types of Haar-like feature patterns. The sum of the pixels that exist within the gray polygon is subtracted from the sum of the pixels in the black polygon. The areas with the dashed lines indicate insignificant pixels.

HOG features [7] are the most popular features used for human detection and have a lower false-positive rate. However, high computational demands are a drawback of HOG. To produce compact feature patterns, we first extract the Haar-like features [8] (differences in the rectangular sums) from integral images. For Haar-like features, we designed 27 types of features, as shown in Figure 2, by considering the symmetry of the human body. Next, 27 types of Haar-like patterns are concatenated to produce one Haar-like descriptor with 27 dimensions. Although increasing the Haar-like patterns improves the performance, the run-time cost depends on the feature dimensions. In our study, we set the proper number of Haar-like patterns to 27 according to the experimental results.

As the second feature, we use an oriented center-symmetric local binary pattern (OCS-LBP) [10] feature because it supports the gradient magnitude and pixel orientation.

### 3.2. Cascade Random Forest

**Figure 3.**
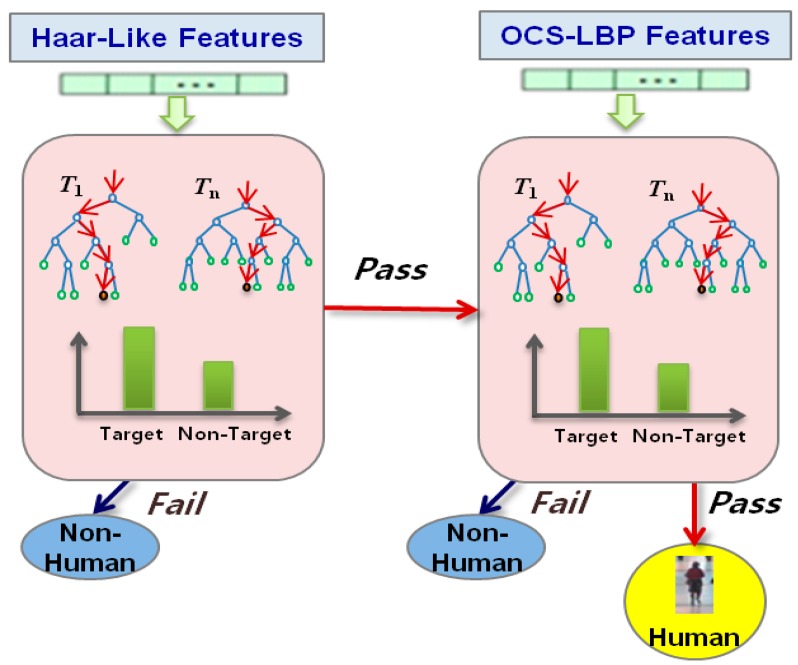
The structure of the two-level CaRFs using Haar-like features and OCS-LBP descriptors. If the candidate window passes through two random forests, it is declared as the final human region.

After selecting the image-scaling levels with their adaptive ROI, we employ a CaRF classifier by modifying the works in [2,9] to separate candidate windows into both human and non-human classes. CRF is a combination of a series of random forest classifiers as a filter chain, as shown in Figure 3. A random forest is a decision tree ensemble classifier, where each tree is grown using some form of randomization [12]. A random forest has the capacity for processing huge amounts of data with high training speeds based on a decision tree. Each filter is a set of strong classifiers (decision trees) consisting of a number of n weak classifiers (split functions). When the test image is used as input to the trained random forest, the final class distribution is generated by an ensemble (arithmetic averaging) of all tree distributions *L* = (*l*_1_, *l*_2_,…, *l_T_*), and we choose *c_i_* as the final class (*f*) of the input image if the final class distribution *p*(*c_i_* | *L*) has the maximum value:

(2) $$f=\arg\underset{i=1\;\text{to}\;N}{\max}\left\{\frac{1}{T}\sum_{t=1}^{T}P\left(c_{i}|l_{t}\right)\right\}$$

The important parameters of a random forest are the tree depth and number of trees, *T*. We set the maximum tree depth to 20, and the number of tree sets to 80 for the first level and 100 for the second level, according to the experimental results.

For this paper, we generate two-level CaRFs using Haar-like features for the first random forest, and OCS-LBP for the second random forest. From a two-level CaRF, we can increase the detection accuracy by removing negative windows at each level, which allows human detection to be conducted in real-time.

## 4. Experimental Results

**Figure 4.**
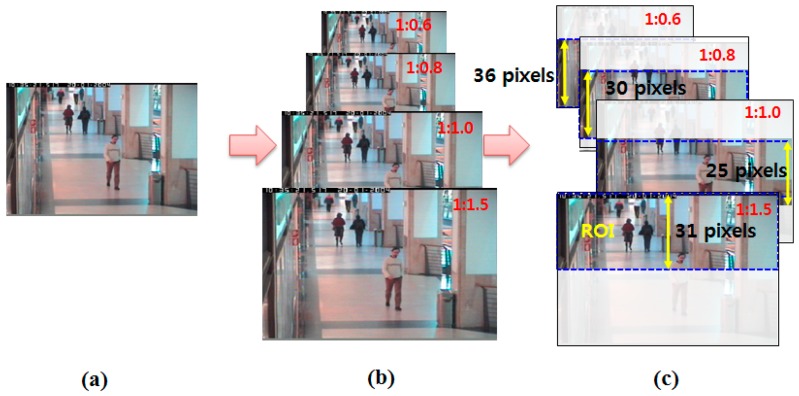
The selected image-scaling levels and ROI size per scaling level: (**a**) original image; (**b**) four image-scaling levels; and (**c**) ROI size (pixels) for each scaling level.

We assessed the performance of our proposed fast human detector using the CAVIAR [13] and PETS2009 [14] datasets. The CAVIAR dataset consists of twenty video sequences at a resolution of 384 pixels × 288 pixels and 25 frames per second (fps). The PETS2009 dataset consists of one video sequences, also with a resolution of 384 pixels × 288 pixels and 25 fps. Among the few available public datasets, we selected the CAVIAR and PETS2009 datasets because their images were captured from a camera installed at a high position. Experiments on human detection from the test data were conducted using an Intel Core i-7 Quad processor PC running Windows 7 OS. To estimate the scaling level using an adaptive ROI and training of the CRF, we used five CAVIAR video sequences including 13,282 frames. From the proposed HWM with a divide-and-conquer algorithm, we estimated four of eleven scaling levels: the original size, up-sampling at a ratio of 1:1.5, down-sampling at a ratio of 1:0.8, and 1:0.6, as shown in Figure 4. Figure 4 also shows the adaptive sizes (pixels) of the ROIs and their position according to the image-scaling levels. For example, the size of the human is larger than in the other regions when the human stands near the camera. In this case, the image is down-sampled at a ratio of 1:0.6, and the ROI at the front region is set to detect large-sized humans. Moreover, the ROIs of all scaling levels are allowed for overlapping between the ROIs to prevent missing humans located within the ROI boundary.

The CaRF classifier was trained using 12,058 positive training samples and 7156 negative examples sampled randomly from background images containing no humans, at a size of 30 pixels × 69 pixels. For a comparison of the human detection performance, we evaluated both the false positives per image (FPPI) and the recall. We compared the proposed algorithm with other related methods [3,4,6,7] using fifteen CAVIAR video sequences including 16,741 frames, and the first 795 frames from Scenario S2.L1 of the PETS2009 dataset. To evaluate the performance of the proposed scaling algorithm for human detection, we compared it with four other scaling algorithms:

- Method 1: Densely sampled image pyramid [7].
- Method 2: Sparsely sampled image pyramid [4].
- Method 3: Sparsely sampled pyramid with static ROI [6].
- Method 4: Scaling the features not in the image [3].

**Figure 5.**
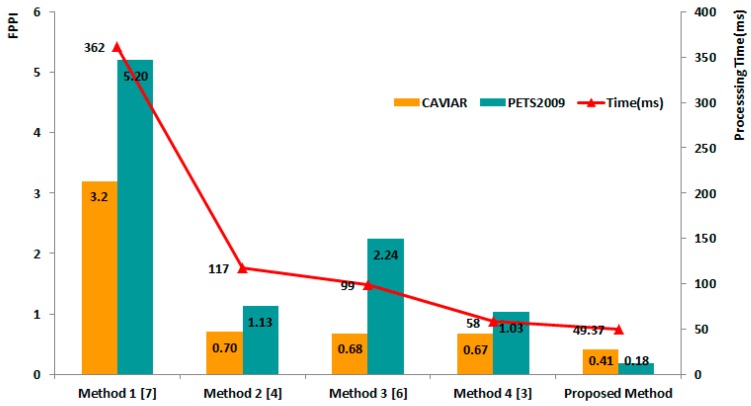
Performance comparison of FPPI and the computational time for the proposed method versus four other methods using different image-scaling techniques. The average FPPIs and recall of the detection results for Methods 2 and 3 were performed in the same condition of [4] and [6]. For Method 1, we used the source code of OpenCV. In addition, we used the open-source of Benenson *et al.* [3] for method 4.

First, we evaluated the FPPI performance using both the CAVIAR and PETS2009 datasets. Figure 5 shows a FPPI comparison of the results of the five different methods. Overall, we confirmed that our proposed algorithm produces the lowest FPPI results compared with the other four methods for the two datasets. In particular, the proposed method shows a 5.02 lower FPPI than Method 1, which uses a multi-scale image pyramid on the PETS2009 dataset. In the case of the CAVIAR dataset, the four other methods showed similarly low FPPI results, with the exception of Method 1. However, our method still showed a 0.26 lower FPPI than Method 4, which had the lowest FPPI among the four comparison methods. The processing time of the proposed method (49 ms per image) was faster than that Method 1 (362 ms per image), Method 2 (117 ms per image), Method 3 (99 ms per image), and Method 4 (58 ms per image). Although Method 4 showed a rate of 20 ms per image in [2] when using both the CPU and GPU concurrently, it provided only 58 ms per image when we tested its performance using the same system environment with only a CPU.

**Figure 6.**
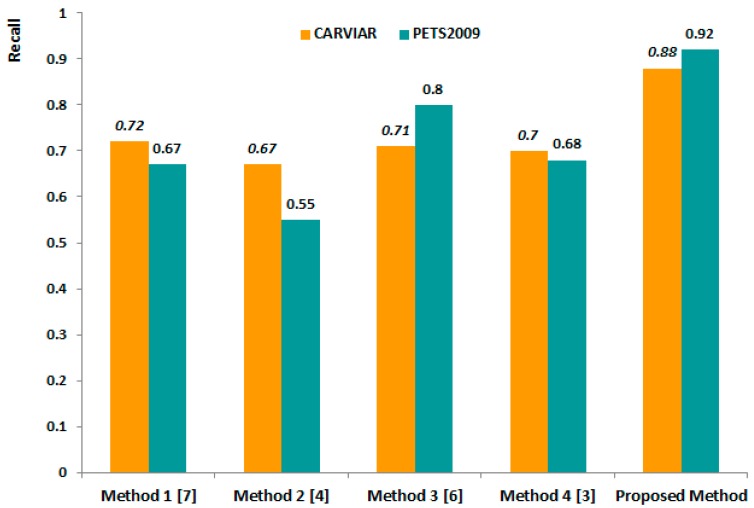
Performance comparison of the proposed method and the four other methods using different image-scaling methods.

Second, we compared the recall performance using the same CAVIAR and PETS2009 datasets. The recall rate of the proposed method outperformed the other methods at 0.88 *vs.* 0.72, 0.67, 0.71 and 0.7, respectively, when we used the CAVIAR datasets, as shown in Figure 6. When we used the PETS dataset, the recall difference between the lowest rate of Method 2 and proposed method is larger than the difference for the CAVIAR dataset, *i.e.*, 0.37 *vs.* 0.21.

As can be seen from the overall results, the proposed algorithm shows a fast and good detection performance for the following reasons.

1. The proposed method applies a human detector to only sparsely selected image scales.
2. The adaptive ROIs per image scale limit the range of the scanning window.
3. CaRF increased the detection accuracy by removing false windows at each level using a cascade method.

**Figure 7.**
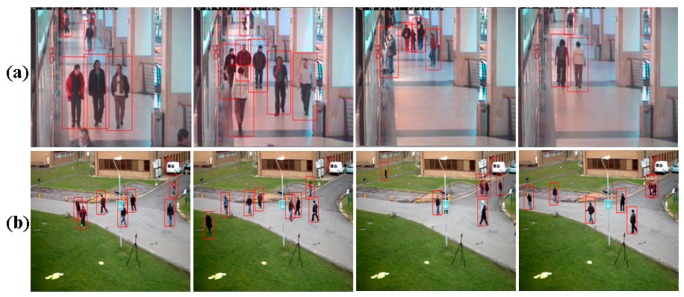
Sample human detection results of the proposed method using the (**a**) CAVIA and (**b**) PETS2009 test datasets.

Figure 7 shows some human detection results from our proposed method using the CAVIAR and PETS2009 test datasets. As shown in Figure 7, our proposed method detected humans correctly in the test video sequences containing humans of different sizes.

## 5. Conclusions

We have demonstrated that HWM with a divide-and-conquer algorithm provides the optimal levels of image scaling for human detection in surveillance video sequences. Moreover, an adaptive ROI for image scaling helps improve the detection accuracy and reduce the detection time. We also proved that CaRF based on Haar-like features and an OCS-LBP descriptor exhibits distinct patterns for human detection and is a suitable descriptor for distinguishing humans from background objects when used together with a CaRF classifier. In the future, we plan to improve our algorithm to reduce the processing time, and allow the articulated deformations of humans to be handled in video sequences for real-life surveillance applications.
